# Use of herbal products by diabetic patients in coping with health problems: a cross-sectional study from Turkey

**DOI:** 10.1007/s11739-025-03938-y

**Published:** 2025-04-14

**Authors:** Elif Doğar, Hatice Tuba Akbayram, Yıldız Büyükdereli Atadağ

**Affiliations:** Department of Family Medicine, Faculty of Medicine, Gaziantep University, 27600 Gaziantep, Turkey

**Keywords:** Diabetes mellitus, Traditional and complementary medicine, Herbal product

## Abstract

This study aimed to determine the herbal products used by type 2 diabetes patients for health problems, the purpose of use, and related factors. This cross-sectional study employed a face-to-face interview with patients who had applied to the diabetes outpatient clinic between June–October 2022. The questionnaire included questions about personal characteristics, data related to disease and treatment, the herbal products used, and their intended use. The mean age of the 280 patients was 58.18 ± 9.4 years, with 63.9% female. A total It 30.4% patients reported the use of herbal products. Those with a high school education or above used it significantly more than those with less education (p = 0.014). No significant relationship was found between herbal product use and diabetes duration, insulin use and glycated hemoglobin (HbA1c) levels. Patients with nephropathy used herbal products at a significantly lower rate compared to those without nephropathy (p = 0.045). The main reasons for using herbal products were determined as regulating blood sugar (58.8%), losing weight (17.6%), lowering cholesterol (14.1%) and lowering blood pressure (9.4%) and other reasons. Most frequently used herbs were olive leaf, cinnamon, black seed for regulate blood sugar; walnut juice, lemon for hyperlipidemia; garlic, lemon for hypertension, lemon, green tea for weight problem. Approximately one-third of type 2 diabetic patients used herbal products for managing blood glucose, weight, cholesterol, and blood pressure. Education level and the presence of nephropathy were significant factors influencing herbal product use.

## Introduction

Diabetes mellitus is a globally prevalent condition characterized by hyperglycemia, associated with numerous vascular and non-vascular complications [1]. Commonly observed complications include coronary heart disease, stroke, peripheral artery disease, nephropathy, retinopathy, and peripheral neuropathy [2]. It is reported that approximately one in eleven adults worldwide has diabetes, with the majority (90%) having type 2 diabetes [3]. Patients with type 2 diabetes frequently present with cardiovascular risk factors such as hypertension, dyslipidemia, and obesity. In order to effectively manage this disease, it is essential to control risk factors including body weight, blood pressure, blood lipids, and blood glucose through lifestyle modifications [4, 5].

The prevalence of diabetes has risen significantly both globally and in Turkey in recent years [6]. It is estimated that 537 million individuals aged 20–79 have diabetes worldwide, with the number projected to reach 783 million by 2045 [7]. Diabetes is also a significant public health issue in Turkey, with approximately 7 million adults aged 20–79 diagnosed with diabetes in 2020, representing around 15% of the adult population [8].

Due to the chronic nature of diabetes, its lifelong need for dietary and pharmacological treatment, the limitations of modern medical therapies, and the side effects of medications, many patients turn to complementary or alternative treatments. Herbal products are frequently chosen due to perceived fewer side effects and traditional perspectives [9, 10].

Herbal medicine is the most commonly used form of complementary and alternative medicine among diabetic patients worldwide [11]. Approximately 400 medicinal plants have been identified for use in diabetes treatment, though only a small portion has undergone scientific and medical evaluation [12]. The integration of herbal medicine into modern medical practice is hampered by the lack of clinical evidence regarding its safety and efficacy. The lack of large randomized controlled trials comparing herbal therapies with existing oral hypoglycemic agents, possible side effects, drug interactions, and problems with product standardization are the main concerns associated with the use of herbal products [13].

Diabetic patients often use herbal products not only for blood glucose control but also for weight loss, cholesterol reduction, blood pressure management and diabetes complications. Although many studies have been conducted on the use of herbal products by diabetic patients, there is limited information specifically examining the intended purposes and the types of herbs used. This study aims to determine the herbal products used by patients with type 2 diabetes, their intended purposes, and related factors**.**

## Methods

### Study design and sample size

This cross-sectional was conducted with patients who applied to the diabetes outpatient clinic of Gaziantep University Faculty of Medicine Hospital between 01/06/2022–01/11/2022. Patients were informed about the study. The patients who agreed to participate in the study were interviewed face to face and the questionnaire form was applied.

Inclusion criteria were as follows: outpatient admission to the diabetes outpatient clinic, consent to participate in the study, being 18 years of age or older, having been diagnosed with type 2 diabetes at least one year ago, being able to speak Turkish, not being a pregnant or nursing mother, not having cognitive dysfunction and not having a serious psychiatric disorder such as schizophrenia. Exclusion criteria were defined as giving incomplete answers to the survey questions or being diagnosed with type 1 diabetes.

### Questionnaire form

The 29-item questionnaire, developed by the researchers through a literature review, was divided into three sections. The first section included questions on the socio-demographic characteristics of the participants, such as age, sex, education level, marital status, and income. The second section included questions about the duration of diagnosis, diet and exercise, medications used, presence of comorbidities, complications and glycated hemoglobin (HbA1c) values measured in the last three months. HbA1c values were obtained from electronic patient files with the consent of the patients. In the third section, patients were asked about their use of herbal products in the last year and the purposes for which these products were used. In addition, open-ended and multiple-choice questions were included about the herbal products used to regulate blood sugar, lose weight, lower blood pressure or reduce cholesterol, where this information was obtained and whether it was reported to the doctor, and perceived benefits and harms. The questionnaire was pretested in a pilot study of 15 patients whose data were not included in the study. Based on participant feedback, some questions have been made easier to understand.

### Ethics

Approval was obtained from the Gaziantep University Clinical Research Ethics Committee (decision dated 07/02/2022 and numbered 2022/15) before the study started. All patients participating in the study were informed and their verbal and written informed consent was obtained.

### Statistical analysis

The data were analyzed using the SPSS 22.0 program. While numbers and percentages were employed in descriptive statistics, a Pearson Chi-square test was utilized to compare analytical data. The conformity of the numerical data to a normal distribution was evaluated using the Shapiro–Wilk test. For non-normally distributed data, a Mann–Whitney U test was employed for two-group comparisons. A p-value of less than 0.05 was considered statistically significant.

## Results

The mean age of the 280 patients included in the study was 58.18 ± 9.4 years and 63.9% were female. It was found that 84.3% of the patients were married, 61.8% had primary education, 58.2% had moderate income, 63.6% paid attention to their diet most of the time and 54.3% did not exercise (Table 1).

**Table 1:** Descriptive characteristics of patients

Characteristics		n	%
Age mean ± Standard deviation (years): 58.18 ± 9.42			
Age (years)	< 65	211	75.4
	≥ 65	69	24.6
Sex	Male	101	36.1
	Female	179	63.9
Marital status	Married	236	84.3
	Not married	44	15.7
Education level	Illiterate	69	24.6
	Elementary education	173	61.8
	High School	26	9.3
	University	12	4.3
Economic situation	Low	97	34.6
	Moderate	163	58.2
	High	20	7.1
Watch your diet	Rarely	40	14.3
	Sometimes	62	22.1
	Most of the time	178	63.6
Exercise	Does not	152	54.3
	Irregular	62	22.1
	Regular*	66	23.6

^*^At least half an hour of moderate exercise (e.g. walking) most days of the week

The mean HbA1c value of the participants was 8.40 ± 2.18 and 65.7% was 7% or higher. 37.1% of them were using insulin and oral antidiabetic drugs together as treatment. It was found that 59.3% of the patients had hypertension, 49.3% had hyperlipidemia, 32.5% had cardiovascular disease, 18.2% had diabetic foot, 23.9% had retinopathy, 14.6% had nephropathy, and 51.1% had neuropathy. Some characteristics of the patients related to diabetes are shown in Table 2.

**Table 2:** Diabetes disease characteristics of the patients

Characteristics		n	%
Diabetes diagnosis time (years)	< 10	154	55
	≥ 10	126	45
Diabetes treatment	Only one oral antidiabetic	64	22.9
	More than one oral antidiabetic	84	30
	Insulin only	28	10
	Insulin and oral antidiabetics	104	37.1
HbA1c (%)	< 7	96	34.3
	≥ 7	184	65.7
Hypertension	Yes	166	59.3
	No	114	40.7
Hyperlipidemia	Yes	138	49.3
	No	142	50.7
Cardiovascular disease	Yes	91	32.5
	No	189	67.5
Diabetic foot	Yes	51	18.2
	No	229	81.8
Retinopathy	Yes	67	23.9
	No	213	76.1
Nephropathy	Yes	41	14.6
	No	239	85.4
Neuropathy	Yes	143	51.1
	No	137	48.9

It was determined that 30.4% (n = 85) of the patients had used herbal products for treatment purposes in the past year. When the relationship between the use of herbal products and patients'age categories, sex, marital status, income level, diet, and exercise habits was analyzed, no statistically significant association was found (p > 0.05). However, the rate of herbal product use among individuals with a high school or university education (47.4%) was significantly higher compared to those with lower education levels (27.7%) (p = 0.014).

Diabetes disease characteristics of the patients and their use of herbal products were compared. It was found that the rate of herbal product use in patients with nephropathy (17.1%) was statistically lower than that in patients without nephropathy (32.6%) (p = 0.045) The relationship between the disease characteristics of the participants and herbal product use is shown in Table 3.

**Table 3:** Relationship between some characteristics of patients and herbal product use status

Variable	Categories	Herbal product users (n = 85)	Not using herbal products (n = 195)	p
Age (years)	< 65	70 (% 33.2)	141(% 66.8)	0.073
	≥ 65	15 (% 21.7)	54(% 78.3)	
Sex	Male	29 (% 28.7)	72 (% 71.3)	0.653
	Female	56 (% 31.3)	123 (% 68.7)	
Marital status	Married	73 (% 30.9)	163 (% 69.1)	0.628
	Not married	12 (% 27.3)	32 (% 72.7)	
Education level	Under high school	67 (% 27.7)	175 (% 72.3)	**0**.**014**
	High school and above	18 (% 47.4)	20 (% 52.6)	
Economic situation	Low	27 (% 27.8)	70 (% 72.2)	0.631
	Moderate	53 (% 32.5)	110 (% 67.5)	
	High	5 (% 25)	15 (% 75)	
Watch your diet	Rarely	15 (% 37.5)	25 (% 62.5)	0.164
	Sometimes	23 (% 37.1)	39 (% 62.9)	
	Most of the time	47 (% 26.4)	131 (% 73.6)	
Exercise	Does not	41 (% 27.0)	111 (% 73.0)	0.149
	Irregular	25 (% 40.3)	37 (% 59.7)	
	Regular	19 (% 28.8)	47 (% 71.2)	
Duration of diabetes (years)	< 10	48 (% 31.8)	106 (% 68.8)	0.744
	≥ 10	37 (% 29.4)	89 (% 70.6)	
Insulin treatment	Yes	41 (% 31.1)	91 (% 68.9)	0,809
	No	44 (% 29.7)	104 (% 70.3)	
HbA1c level (%)	< 7	26 (% 27.1)	70 (% 72.9)	0.389
	≥ 7	59 (% 32.1)	125 (% 67.9)	
Hypertension	Yes	48 (% 28.9)	118 (% 71.1)	0.527
	No	37 (% 32.5)	77 (% 67.5)	
Hyperlipidemia	Yes	45 (% 32.6)	93 (% 67.4)	0.419
	No	40 (% 28.2)	102 (% 71.8)	
Cardiovascular disease	Yes	23 (% 25.3)	68 (% 74.7)	0.199
	No	62 (% 32.8)	127 (% 67.2)	
Diabetic foot	Yes	11 (% 21.6)	40 (% 78.4)	0.131
	No	74 (% 32.3)	155 (% 67.7)	
Retinopathy	Yes	17 (% 25.4)	50 (% 74.6)	0.309
	No	68 (% 31.9)	145 (% 68.1)	
Neuropathy	Yes	43 (% 30.1)	100 (% 69.9)	0.915
	No	42 (% 30.7)	95 (% 69.3)	
Nephropathy	Yes	7 (% 17.1)	34 (% 82.9)	**0.045**
	No	78 (% 32.6)	161 (% 67.4)	

The bold value indiactes the p < 0.05 was accepted statistically significant

It was determined that 58.8% (n = 50) of the patients used herbal products to regulate their blood sugar levels. The HbA1c value of patients who used herbal products to regulate blood sugar (median 8.7%) was found to be statistically significantly higher than the HbA1c value of patients who did not use herbal products to regulate blood sugar (median 7.7%) (p = 0.028). In addition to regulating blood glucose, patients were found to utilize herbal products for weight loss (17.6%), reducing cholesterol (14.1%), lowering blood pressure (9.4%), and other reasons (23.5%). The reasons for which patients employed herbal products are illustrated in Table 4.

**Table 4:** Reasons for patients'use of herbal products* (n = 85)

Reasons	n	%
Regulating blood sugar	50	58.8
Reduce cholesterol	12	14.1
Reducing blood pressure	8	9.4
Lose weight	15	17.6
Other health problems	20	23.5

^*^Some patients stated more than one reason

The findings revealed that diabetic patients mainly used olive leaves, cinnamon, black cumin, lemon, ginger, thyme, turmeric and sumac to regulate blood sugar. The herbal products used by diabetic patients to regulate blood sugar are presented in Table 5.

**Table 5:** Herbal products used by patients to regulate blood sugar* (n = 50)

Product Name	n	%
Olive leaf	15	30
Cinnamon	13	26
Black cumin	9	18
Lemon	7	14
Ginger	7	14
Thyme	7	14
Turmeric	6	12
Sumac	5	10
Herbal mixture	4	8
Green tea	3	6
Fig leaf	3	6
Clove	2	4
Mulberry leaf	2	4
Vinegar	2	4
Herbal medicine	2	4
Garlic	1	2
Sage	1	2
Mint	1	2
Bay leaf	1	2
Parsley	1	2
Pomegranate syrup	1	2
Linden	1	2
Chamomile	1	2
Centaury oil	1	2
Shepherd's tea	1	2

^*^Some patients specified more than one product

The most commonly used herbal products are walnut juice, lemon, green tea to lower cholesterol, garlic, lemon to lower blood pressure, lemon, lemon and green tea for weight loss (Table 6).

**Table 6:** Herbal products used to reduce cholesterol, lower blood pressure and lose weight*

Products used to lower cholesterol (n = 12)	n	%
Walnut water	8	66.6
Lemon	2	16.6
Green tea	1	8.33
Parsley	1	8.33
Garlic	1	8.33
Sumac	1	8.33
Pomegranate molasses	1	8.33
Products used to lower blood pressure (n = 8)		
Garlic	3	37.5
Lemon	3	37.5
Sumac	1	12.5
Sandarac gum	1	12.5
Unknown product	1	12.5
Products used for weight loss (n = 15)	n	%
Lemon	6	40
Green tea	3	20
Mixed herbal tea	2	13.3
Slimming tea	1	6.6
Parsley	1	6.6
Vinegar	1	6.6
Ginger	1	6.6
Okra seeds	1	6.6
Cinnamon	1	6.6
Mint	1	6.6
Black cumin	1	6.6
Unknown product	1	6.6

^*^Some patients specified more than one product

It was found that 28.2% (n = 24) of the patients who used herbal products used herbal products daily, while 71.8% of the majority did not use herbal products daily. When the patients who used herbal products were asked whether they changed their medication use while using the products, it was found that two patients (2.4%) stopped using medication and six patients (7.1%) reduced the frequency of medication use.

The sources of information about herbal products were relatives/friends (56.5%), internet (18.4%), health professionals (11.8%), and other (newspapers, magazines, books) (4.7%). While 7.1% (n = 6) of the patients who used herbal products stated that they had experienced any harm related to the product, 55.3% (n = 47) stated that it was beneficial.

## Discussion

The findings of this study indicate that patients with diabetes widely use herbal products as a complementary approach to managing various health problems. To the best of our knowledge, this is the pioneering study to investigate the herbal products used by diabetic patients to manage hyperlipidemia, hypertension, overweight issues, and hyperglycemia. It is therefore anticipated that the findings of this study will contribute to the existing literature on the subject. The findings of our study indicate that approximately one-third (30.4%) of patients with type 2 diabetes utilized herbal products for therapeutic purposes over the past year. This rate is similar to the findings of recent studies conducted in Turkey that have documented the prevalence of herbal product use among patients with diabetes, with rates of 36.5% and 36.7%, respectively [14, 15]. The usage of herbal products/drugs by diabetic patients has been documented at rates of 68% in Saudi Arabia, 37.5% in Thailand, and 51.9% in Palestine [16–18]. It is probable that discrepancies in the study population and methodology are responsible for the observed discrepancies in the results of these studies.

The findings of this study show that patients using herbal products mostly use herbal medicines to regulate blood sugar levels. Among these, olive leaf, cinnamon and black cumin seed are the most frequently used ones.

In this study, it was determined that herbal products were primarily used to regulate blood sugar levels, and olive leaves, cinnamon and black cumin were most frequently used for this purpose. Mekuria et al. [9] found that type 2 diabetes patients in Ethiopia mostly used garlic (Allium sativum L.), Giesilla (Caylusea abyssinica), and Tinjute. One literature review was determined that the most commonly of Saudi diabetic patients used products were fenugreek, black seed and neem (Melia azedarach) [11]. Studies conducted in Turkey, consistent with our findings, have shown that patients most frequently use cinnamon, olive leaf, black seed (Nigella sativa L.), and black seed oil for diabetes treatment [14, 19]. The variations in results across countries may be attributed to differences in dietary habits and cultural characteristics.

The present study revealed that HbA1c levels were significantly elevated in patients who utilized herbal products for the purpose of regulating blood glucose compared to those who did not. This finding is consistent with studies conducted in Ethiopia and Kuwait [20, 21]. This may be due to patients who struggle to achieve glycemic control with conventional medications resorting more frequently to herbal products, or due to inconsistent and insufficient use of antidiabetic drugs among herbal product users. Indeed, in our study, 2.4% of patients using herbal products had discontinued their medication, and 7.1% had reduced the frequency of their medication use.

The study found that patients most commonly used walnut water and lemon to lower cholesterol, garlic and lemon to reduce blood pressure, and lemon and green tea for weight loss. Similarly, it was reported that the herbal products most commonly used by hypertensive patients in Sierra Leone were honey, moringa and garlic, while ginger, lemon and garlic were used in Jamaica [22]. Tulunay et al. [23] determined that lemon and garlic are most commonly used for lowering blood pressure, cinnamon for diabetes and walnut for hyperlipidemia in Turkey. These data show that herbal treatment methods for health problems vary in different geographies, but garlic and lemon are universally used.

Our study found that patients with a high school education or higher used herbal products more often than those with less education. In addition, the rate of herbal product use in patients with nephropathy (17.1%) was statistically lower than that in patients without nephropathy (32.6%). Mekuria et al. [9] showed that higher educational status and the presence of diabetes complications increased the likelihood of herbal product use. However, diabetes complications were not analyzed separately in the study of Mekuria et al. [9]. The lower use of herbal products in patients with nephropathy in our study may be due to the fact that patients with renal dysfunction are a sensitive risk group regarding herbal use and that the patients were informed about this issue.

It is notable that in this study, the primary source of information about herbal products was relatives or friends. Furthermore, the majority of patients (74.1%) did not inform their physicians that they used herbal products. These results are in line with many studies [15, 16, 23]. However, given the potential interactions between medical treatment and herbal products, this may pose serious health risks [24]. Therefore, it is important for physicians to ask diabetic patients about the use of herbal products and to inform them about possible side effects.

The strengths of this study are that it provides a detailed assessment of the use of herbal products by diabetic patients with multiple health problems and for what purpose. However, there are also some limitations. Firstly, it was conducted at a single center with a limited patient population, which may restrict the representativeness of the sample and, consequently, the generalizability of the findings to the broader diabetic population. Additionally, data on herbal product use were obtained through a self-reported questionnaire, making them susceptible to recall and response biases. Lastly, the cross-sectional design of the study precludes the determination of causal relationships between herbal product use and health outcomes in diabetic patients. Future multicenter studies involving different countries and cultures will provide a more comprehensive understanding of the topic.

## Conclusion

The study revealed that approximately one-third of patients utilized herbal products for coping with health problems. Herbal products have been found to be used not only to regulate blood sugar, but also to lower cholesterol, lower blood pressure, aid weight loss and address other health concerns. Our study also found that HbA1c levels were significantly higher in patients using herbal products compared to those who did not use them. Physicians should manage diabetes and its comorbidities in accordance with clinical guidelines, inquire about herbal product use, and inform patients about their potential risks and interactions.
